# Cooperation With Universities in the Development of Eco-Innovations and Firms’ Performance

**DOI:** 10.3389/fpsyg.2020.612465

**Published:** 2020-11-24

**Authors:** Juan J. Arroyave, Francisco J. Sáez-Martínez, Ángela González-Moreno

**Affiliations:** ^1^ Faculty of Economics and Business Administration, University CES, Medellín, Colombia; ^2^ Faculty of Economics and Business Administration, University of Castilla-La Mancha, Albacete, Spain

**Keywords:** value cocreation, eco-innovation, operational flexibility, performance, cooperation with universities

## Abstract

In recent decades, the expansion of economic activity has been accompanied by negative environmental impacts. In response, there have been dramatic changes worldwide in terms of an increased demand for environmentally friendly products and services. To achieve these eco-innovations, firms have sought to acquire knowledge and implement operational flexibility by cooperating with different agents such as universities through a value cocreation system that is also expected to enhance firms’ performance. Using a sample of 250 companies, the present paper examines the role of cooperation with universities in the development of diverse environmental innovations and building operational flexibility and, through this, improving firm performance. Results show that firms that value cooperation with universities develop a wider range of environmental innovations and increase their sales and benefits.

## Introduction

During the last few decades, the development of the internet and data analysis (Geczy et al., 2014), the abundance of available information (Southwell, 2005), globalization (Mark, 1996), and increased consumer power (Kucuk, 2008), or what is known as the sharing economy (Belk, 2018), have brought about dramatic changes that affect people and organizations (Sobrino et al., 2019), as well as have negative environmental impacts. In response, environmental issues have become a top priority for governments, which, through regulations and fiscal incentives, among others, have been promoting “eco-innovations” (OECD, 2009). These innovations seek to reduce pollution and other negative impacts of economic and business activities on the environment (Kemp and Pearson, 2007).

Moreover, because of increased consumer power and awareness of environmental concerns, consumers are increasingly willing to pay for products or services produced in a more environmentally conscious way (McDonagh and Prothero, 2014). Thus, there is a market pull toward environmental innovations, providing a means for firms to improve their competitive advantage.

However, because most firms lack sufficient knowledge to respond to these expectations on their own, they must cooperate with different agents; as a result, their image extends beyond a traditional image of a supplier that produces goods and services to be offered to customers, to a value cocreation system in which participants integrate their resources and competencies to increase the creation of value in a service system (Vargo et al., 2008; González-Torres et al., 2020).

In the last two decades, the number of theoretical and empirical contributions to the development of eco-innovations has been increasing (González-Moreno et al., 2019). Additionally, since the seminal work of Prahalad and Ramaswamy (2000) and the proposal of a service-dominant logic by Vargo and Lusch (2004), many authors have worked in the field of value cocreation, giving rise to an abundant, and varied literature that stems from overcoming a linear vision of value chains and value creation (González-Torres et al., 2020).

In this sense, eco-innovation and value cocreation are increasingly considered potential strategies to enhance the firm’s competitiveness in international markets and are thus attracting interest from both industry and academia. The role of firm’s cooperation with universities in the development of innovations has been previously studied (Bayona et al., 2001; Lutchen, 2018). Additionally, the literature has highlighted the importance of cooperation in the development of environmental innovations (De Marchi, 2012; Albort-Morant et al., 2018; Tumelero et al., 2019). However, the role of cooperation with universities and research institutions as a way to generate environmental innovations is still underestimated (Díaz-García et al., 2015) and its potential to promote firm performance has not been explored in depth (Mascarenhas et al., 2018). Recently, Jové-Llopis and Segarra-Blasco (2018) studied the relationship between eco-innovation strategies and firm performance in terms of sales growth in a large sample of European small and medium-sized enterprises (SMEs) but did not consider the effect of cooperation. The main goal of this article is to fill this gap by providing an analysis of the effect of cooperation with universities to achieve eco-innovation and enterprise results. We also include operational flexibility in the analysis as a capability that could enhance firms’ performance and be increased by cooperation with universities and research institutions.

The rest of this article is organized as follows. The next section provides a theoretical overview of the topic. Then, we present the methodology, followed by the results and discussion. Finally, the conclusions section presents the limitations and future research directions.

## Theoretical Framework

### Eco-Innovation and Firm Performance

In recent decades, the global economic environment has been characterized by the world has been facing a new environment characterized by its volatility, uncertainty, complexity, and ambiguity (Whiteman, 1998), which has resulted from the expansion of economic activity among other factors and been accompanied by environmental concerns such as climate change, energy security, and the increasing scarcity of resources (OECD, 2009). Hence, sustainability has been a top priority for governments, and many have adopted long-term frameworks to tackle these concerns (OECD, 2009).

In addition, new generations have a greater awareness of such environmental problems, and consumers are willing to pay a higher price for products or services produced in a more environmentally friendly way (McDonagh and Prothero, 2014). Therefore, the expectations for greater industry efforts to achieve sustainable development have been increasing, and the sustainable manufacturing of new products and services in an environmentally friendly manner has been at the heart of industry policy and practices this century (Triguero and González-Moreno, 2019).

In this sense, stakeholders recognize that sustainable manufacturing has become a key to improving financial results at the firm level, given that environmental concerns drive the generation of competitive advantages (Díaz-García et al., 2015). Hence, firms need to innovate in order to be more efficient and focus on the development of new products or processes that are environmentally friendly; this is known as “eco-innovation” and can be defined as: *“The production, assimilation or exploitation of a product, production process, service or management or business method that is novel to the organization (developing or adopting it) and which results, throughout its life cycle, in a reduction of environmental risk, pollution and other negative impacts of resources use (including energy use) compared to relevant alternatives”* (Kemp and Pearson, 2007, p. 8).

As mentioned above, eco-innovation, which is also known as “green innovation” and “environmental innovation,” has become a market pull owing to consumer demand for greener products and services (Kesidou and Demirel, 2012).

The use of cleaner technologies reduces the likelihood of costs associated with environmental risks (Shrivastava, 1995) and may also contribute to the reduction of manufacturing costs (Christmann, 2000). Moreover, because environmental concerns have been a top priority for governments, regulations, and fiscal incentives have been used as effective drivers of eco-innovation to enable firms to implement environmental regulations that improve their performance (Triguero et al., 2013). Additionally, eco-innovation can lead to the improvement of a firm’s reputation (Moreno-Mondéjar et al., 2020) for being environmentally sound, which may enhance its reputation for quality.

Literature has demonstrated how eco-products (Kammerer, 2009), eco-processes (Hojnik and Ruzzier, 2016), and recycling of waste or materials (Doran and Ryan, 2016) positively and separately influence firms’ performance. Additionally, recent empirical evidence also suggests that the existence of complementarity between different types of eco-innovations and that this increases firm performance (Moreno-Mondéjar et al., 2020). Firms that implement two or more types of complementary eco-innovations will obtain better results than those applying only one of them (Cainelli et al., 2011) because there might be interdependence between, for example, product and process eco-innovation (Cheng et al., 2014).

Therefore, it is possible to expect a positive relationship between the diversity of eco-innovation strategies and firm performance. Formally, we propose that:

Hypothesis 1: The implementation of a variety of eco-innovations positively influences firm performance.

### Cooperation With Universities and Performance

In accordance with the discussion above, it is crucial to be aware that in order to adopt eco-innovations or, indeed, any kind of innovation, technology-push drivers are decisive in explaining these adoptions; therefore, eco-innovations depend on a firms’ technological capabilities, which determine the probability of eco-innovation (Horbach et al., 2013).

Additionally, it is considered that research and development (R and D) increases the degree of innovativeness of a firm with regard to eco-innovations (Tumelero et al., 2019). However, most firms lack resources to invest in internal R and D and, in this sense, most of them do not have the necessary knowledge to eco-innovate on their own. Hence, to innovate beyond their own limits, firms need to establish alliances with external agents such as suppliers, clients, and research centers, in order to develop their capability for innovation (De Marchi, 2012; Albort-Morant et al., 2018; González-Moreno et al., 2019).

Thus, such cooperation with external agents enables firms to evolve from a traditional supplier that produces goods and services to be offered to customers to a value cocreation system in which participants integrate their resources and competencies to increase the creation of value in a service system (Vargo et al., 2008; González-Torres et al., 2020).

Therefore, association with other agents has turned to innovation (Sobrino et al., 2019), challenging traditional concepts by encouraging firms to break with conventions and existing thought patterns through open innovation in which companies integrate both internal and external knowledge flow. This motivates internal innovation as well as enabling firms to seek out external channels in order to commercialize outcomes with the core idea of integrating knowledge, skills, and ideas from the public (Chesbrough, 2003a,b).

In this sense, universities, as institutional sources specializing in basic research, are one of the main key partners in enhancing eco-innovations (Cainelli et al., 2012). In the last few decades, there has been an explosion in the number of research agreements between firms and universities, facilitating, on one hand, the ability of firms to enhance their own research performance by providing them access to the best scientific and engineering minds, turning them into essential allies in R and D (Lutchen, 2018).

On the other hand, universities have also been receptive to these research alliances because of the challenges in obtaining government support for academic research and, at the same time, because of new academic demands that force them to extend their traditional missions of teaching and research to a third mission that calls on them to contribute more to their local economies more effectively through cooperation with industry (Giuliani and Arza, 2009; Lutchen, 2018).

Universities and research centers have thus turned into essential partners for firms that wish to gain new knowledge for innovation. Universities are involved in scientific production and make a 2-fold contribution to innovation: introducing knowledge and technological staff to increase skills and provide ideas through research that may prove crucial to industry and the innovation process (Bayona-Sáez et al., 2001).

Therefore, universities and research centers are important agents in efforts to achieve eco-innovation because of their crucial role in the innovation system and ability to offer firms basic knowledge. Furthermore, they are a key agent for eco-innovations because, through alliances, both firms and universities may be able to obtain more funds to conduct research projects; such cooperative relationships make it easier to participate in programs for the promotion of innovation, financed by various administrative bodies (Bayona-Sáez et al., 2001). Hence, both universities and industry, through cooperation, will be able to cocreate value and, because of the sum of the inflow and outflow of this knowledge, develop environmental innovations. Formally, we propose that:

Hypothesis 2: Cooperation with universities positively influences the development of eco-innovations.

Flexibility is a strategy to cope with dynamic environments (Gerwin, 1993). The strategic effectiveness of an organization depends on the compatibility of structures and processes within the firm and within the environment in which it operates. Thus, firms should use different strategies to cope with turbulence through operational flexibility, which is understood as the organization’s ability to meet an increasing variety of customer expectations while keeping costs, delays, organizational disruptions, and performance losses at or near zero (Zhang et al., 2002). In this sense, operational flexibility includes the ability to make rapid, low-cost changeovers, adjust capacity incrementally, and quickly launch products with incremental changes within certain parameters in response to market needs.

However, most firms do not have the necessary capabilities to cope on their own with these dynamic environments, where eco-innovations, among other innovations, require that firms to extend their traditional systems and develop strategies that provide the right kind of flexibility to succeed (Moreno-Mondéjar et al., 2020). As a result, most firms need to outsource crucial components and forge supply chain partnerships with other agents (Anand and Ward, 2004). In this sense, cooperation with universities is crucial for operational flexibility, because knowledge acquisition through these alliances empowers the value chain, enabling firms to specialize in their core business by leaving research, which is not their main strength, to the universities and research centers that are experts in that field (González-Torres et al., 2020).

Therefore, through cooperation with universities, firms may be capable of making rapid changes in product design to a wide range of products, because this cooperation would help them to expand their services to meet an increasing variety of customer expectations (Zhang et al., 2002). Hence, it would be possible to propose, as a third hypothesis, that:

Hypothesis 3: Cooperation with universities increases the operational flexibility of firms.

Firms require to have enough capabilities and knowledge to respond and develop solutions to current dynamic environment demands. Therefore, cooperation with universities has turned crucial to enabling firms to acquire basic knowledge and competitive research, to respond to these new demands, as well as to gain access to networks and at the same time to increase their reputation and thus improve their position in the market (Bayona et al., 2001).

Furthermore, cooperation with universities has turned in an opportunity for firms to obtain funding for research projects, run by administrative bodies, and on another hand, has also turned in an opportunity to carry out a long-term technological strategy, in order to make the most of the opportunities offered to them by the public R and D system (Bayona et al., 2001).

Hence, considering that cooperation with universities and research institutions will increase firm’s operational flexibility and the development of eco-innovations and that we expect a positive relationship between the variety of eco-innovations and firm’s performance, and we could propose a direct and indirect effect of cooperation with universities and performance. Cooperation with universities will increase firm’s knowledge base as well firm’s image and reputation (Bayona et al., 2001). This reputation will also improve for being environmentally sound and develop eco-innovations (Moreno-Mondéjar et al., 2020). Therefore, we can propose that:

Hypothesis 4: Cooperation with universities increases firm performance.

### Operational Flexibility and Performance

As already mentioned, increasing global competition, the acceleration of technological changes and expanding customer expectations create a turbulent environment; in response, firms are forced to increase their flexibility to meet the increasing variety of customer expectations. Therefore, the operational flexibility of firms plays an important role in effectively achieving a competitive advantage, because it enables firms to respond, in a rapid and cost-effective manner, to specific customer requests (Carlsson, 1989; Gerwin, 1993).

There is an increasing demand for environmentally friendly products and services. In this sense, through operational flexibility, firms may be able to foster changes in product or processes that may conduce to augmenting their chances to reduce the consumption of inputs such as energy or raw materials, developing environmentally-friendly innovations, and contributing to lessen the environmental damage of the firm’s activity (González-Moreno et al., 2019).

Furthermore, since the adoption of eco-innovations depends on firms’ technological resources and capabilities, firms seek for cooperation with partners to reduce uncertainty and share the risks related to eco-innovations (Triguero et al., 2018). Then, operational flexibility turns crucial to enhance eco-innovations, and recent empirical research shows the role of operational flexibility in the development of biofuel technologies and other eco-innovations (Kou and Zhao, 2013). Hence, we can propose that:

Hypothesis 5: Operational flexibility positively drives eco-innovation.

In this sense, operational flexibility allows firms to respond rapidly to changing customer demands with new innovative products and modifications to existing products. Recognizing how industry evolves is a key capability that is positively associated with firm’s performance (Fawcett et al., 1996) and previous research has examined the direct effect of flexibility on performance through reduction of environmental uncertainty (Alpkan et al., 2007).

In addition, operational flexibility may help firms to provide a smooth flow of materials to the manufacturing process and quick delivery to customers. Furthermore, firms are also able to ensure that different groups, from both inside and outside of the organization, may easily coordinate product design, production, and distribution and, thus, take actions quickly to increase value to customers (Zhang et al., 2002).

Because of such operational flexibility, firms can increase the range of products available, offering more personalized products and services and making rapid changes to product design to quickly adjust their manufacturing capacity (Davis, 1993) and therefore achieving high performance. Consequently, because firms, through operational flexibility, can respond quickly to customer needs with high-quality products, innovative designs, and excellent after-sales services, they can build customer loyalty and thus increase market share to ultimately make large profits (Ferdows and De Meyer, 1990; Flynn and Flynn, 1996). We propose that:

Hypothesis 6: Operational flexibility positively drives firms’ performance.

**Figure 1:**
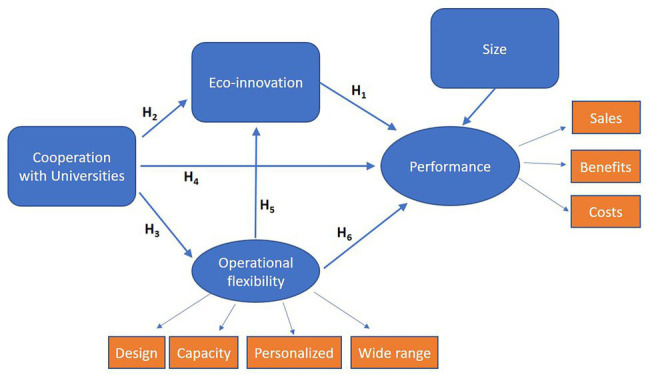
Proposed model and hypotheses.

Figure 1 shows the model and the proposed hypotheses to be compared. We propose that cooperation with universities increases firms’ operational flexibility (H3), thus enhancing firms’ capacity to make rapid changes in product design, to rapidly adjust production capacity, to offer personalized products, and to develop a wide range of products – and, through these increased capabilities – enhances firm performance (H6).

Additionally, cooperation with universities helps firms to cocreate environmental innovations that enable them to reduce the environmental harms caused by their activities (H2). Eco-innovations will also be fostered by operational flexibility (H5). These eco-innovations improve firms’ performance by fostering customer loyalty and even reducing manufacturing costs due to energy and resource consumption. Furthermore, a large variety of eco-innovations will also increase firms’ performance owing to the existence of complementarities between different types of eco-innovations (H1).

Finally, we also expect a direct effect of cooperation with universities and firm performance (H4) and this cooperation will increase the firm’s knowledge base and its reputation.

In our model, we have also included the size of the firm as a control variable. The model demonstrates how we measure performance and operational flexibility. Table 1 gives additional information on variable definition.

**Table 1:** Definition of variables and descriptive statistics.

Variable	Description	Mean	SD	Min	Max
Sales	By how much have your sales increased in the last 3 years compared to your competitors (Likert scale)^α^	3.34	0.71	1	5
Benefits	By how much have your benefits increased in the last 3 years compared to your competitors (Likert scale)^α^	3.29	0.68	1	5
Costs	By how much have your costs reduced in the last 3 years compared to your competitors (Likert scale^α^	3.20	0.74	1	5
Size	Number of employees	133.7	174.4	1	817
Eco-innovations	How many of the following five types of environmental innovation has your company introduced in the last 3 years: eco-product, eco-process, eco-packaging, more ecological distribution channel, and recycling of residuals	1.08	1.07	0	5
Cooperation with Universities	How important is it for your company to cooperate with universities and research institutions for the development of eco-innovations (Likert scale)^β^	0.49	1.61	0	5
Product design	Firm capacity to make rapid changes in product design (Likert scale)^μ^	3.73	1.30	1	5
Production capacity	Firm capacity to rapidly adjust production capacity (Likert scale)^μ^	4.00	1.15	1	5
Personalized products	Firm capacity to offer personalized products (Likert scale)^μ^	3.80	1.28	1	5
Wide range of products	Firm capacity to offer a wide range of products (Likert scale)^μ^	3.84	1.27	1	5

α Takes the value 1 if on the lower 20%; 2 if below average; 3 on average; 4 over average; and 5 on the top 20%.

β Takes the value 0 if no importance/cooperation; 1 if very low importance; 2 if low importance; 3 neutral; 4 if important; and 5 if very important.

μ Takes de value1 if very low capacity; 2 low capacity; 3 neutral; 4 high capacity; and 5 very high capacity.

## Materials and Methods

In order to test our model, we will focus on a particular industry, i.e., the food and beverage industry in Spain. This industry is in the manufacturing sector and accounts for the highest proportion of employment and economic output, both in Spain and in the European Union (Rabadán et al., 2019). The empirical analysis is based on an *ad hoc* survey. Table 1 shows variable definition and descriptive statistics. Questionnaires were distributed in June 2017 to a randomly chosen sample of firms operating in the food and beverage Industry (NACE codes 10 and 11). From a random sample of 1,000 firms, 279 responded to the survey, which represents a 27.9% response rate. Considering the worst possible situation (*p* = *q* = 0.5) for a 95% CI, our margin of error is +/− 5.84%. Finally, 29 cases were eliminated because of omitted data, and our final sample consisted of 250 firms.

In order to test our model and hypotheses, we use EQS software for structural equation modeling (SEM). SEM is a collection of statistical techniques that allows a set of relationships between one or more independent variables and one or more dependent variables to be examined. Both independent and dependent variables can be either continuous or discrete and can be either factors or measured variables (Bentler, 2006). SEM is a general term that covers a variety of statistical models and there are two major approaches to structural equation: covariance-based and variance-based SEM. EQS uses covariance-based SEM, which is the more widely used approach (Astrachan et al., 2014). In this sense, Partial Least Square (PLS) is a variance-based SEM also useful and increasingly applied approach. PLS-SEM has become very popular among social science researchers due to its ability to handle small sample sizes, complex models, and non-normal data distributions (Ringle et al., 2020). Both approaches differ in their basic assumptions and outcomes as well as in their estimation procedures. EQS follows a maximum likelihood estimation procedure, while PLS uses a regression-based ordinary least squares estimation method. However, both try to analyze the cause-effect relations between variables.

As shown in Figure 1, our model comprises two latent variables: performance and operational flexibility. These variables are constructed through three and four items, respectively, and based on extensive literature. Similar subjective performance measures were previously used in Gupta and Govindarajan (1984) and Zhang et al. (2014), among others. Additionally, our operational flexibility variables are based on Anand and Ward (2004).

Table 1 shows the definition of the variables and descriptive statistics.

## Results and Discussion

For testing our model and hypotheses, we used EQS software for SEM. Regarding the goodness of fit of the model, the chi-square is 57.671 with a value of *p* 0.002 and 29 degrees of freedom. The results of the chi-square test were nearly always significant, implying a poor fit of the model to the data, but provided a basis for comparison. Anyway, other measures should be provided (Byrne, 2013). Therefore, we provide other goodness-of-fit tests that indicate that our model fits the data. The X^2^/df ratio is 1.98, below 2.0 (Tabachnick and Fidell, 2007). The CFI (Comparative Fit Index) is 0.982 and NNFI (Bentler-Bonett Non-normed Fit Index) is 0.972, both higher than 0.95, which indicates a good fit (Hu and Bentler, 1999). In addition, the RMSEA (Root Mean Square Error of Approximation) is 0.063, below 0.08, indicating an adequate fit (Browne and Cudeck, 1993). Therefore, considering the values of the global indicators, the overall fit of the model is acceptable.

**Figure 2:**
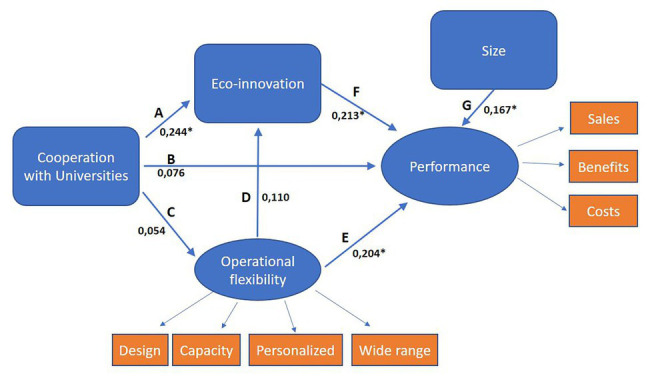
Standardized coefficients. *Significant at 0.05 level.

Concerning our latent variables (performance and operational flexibility), both are explained, and all expected relationships are significant (see Table 2 and Figure 2) Increase in sales (coefficient 0.885) and benefits (coefficient 0.955), and reductions in costs (coefficient 0.781) are the elements that define performance. Similarly, offering a wide range of products (coefficient 0.921) and a capacity to offer personalized products (coefficient 0.935) as well as to make rapid changes in product design (coefficient 0.915) and to rapidly adjust the production capacity (coefficient 0.817) give the firm operational capability. Cronbach alpha of performance (three items) and operational flexibility (four items) are 0.905 and 0.942, respectively. Additionally, Table 2 shows the total effect of the variables as well as its decomposition in direct and indirect effect.

**Table 2:** Decomposition of the parameters of the model.

Pathways between variables	Total effect	Partial indirect effect	Total indirect effect	Direct effect	R-squared
Product design → Oper. Flex.	0.915 (18.713)^*^	-	-	0.915 (18.713)^*^	0.838
Production capacity → Oper. Flex.	0.817 (15.561)^*^	-	-	0.817 (15.561)^*^	0.668
Personalized products → Oper. Flex.	0.935 (19.409)^*^	-	-	0.935 (19.409)^*^	0.874
Wide range products → Oper. Flex.	0.921 (18.909)^*^	-	-	0.921 (18.909)^*^	0.848
Sales → Perform	0.885 (15.768)^*^	-	-	0.885 (15.768)^*^	0.783
Benefits → Perform	0.955 (16.446)^*^	-	-	0.955 (16.446)^*^	0.912
Costs → Perform	0.781 (15.985)^*^	-	-	0.781 (15.985)^*^	0.611
CoopUniv → Oper. Flex.	0.054 (0.826)	-	-	0.054 (0.826) (C)	0.003
CoopUniv → Eco-innov	0.250 (4.066)^*^	CxD = 0.006	0.006 (0.748)	0.244 (3.989)^*^ (A)	0.074
Oper. Flex. → Eco-innov	0.110 (1.744)	-	-	0.110 (1.744) (D)	
CoopUniv → Perform	0.140 (2.176)^*^	AxF = 0.051 CxE = 0.012 CxDxF = 0.001	0.064 (2.527)^*^	0.076 (1.202) (B)	0.141
Oper. Flex. → Perform	0.227 (3.451)^*^	DxF = 0.023	0.023 (1.550)	0.204 (3.160)^*^ (E)	
Eco-innov → Perform	0.213 (3.314)^*^	-	-	0.213 (3.314)^*^ (F)	
Size → Perform	0.167 (2.711)^*^	-	-	0.167 (2.711)^*^ (G)	

Standardized parameter (*t*-value). The letters A, B, C, D, E, F, and G correspond to the notation in Figure 2.

* Significant at 0.05 level.

Our first hypothesis is corroborated. As seen in Figure 2, there is a positive and significant relationship between the diversity of eco-innovations and firms’ performance (coefficient 0.213). Firms that develop a variety of eco-innovations, including eco-products, eco-process, more ecological distribution channels, and eco-packaging and recycling, obtain better results than those focused on a particular type of environmental innovation. Complementarities between the different kinds of eco-innovations would explain this result. Our finding is consistent with recent literature. Cheng et al. (2014) found that eco-product and eco-process innovations complement each other, influencing firm performance. Recently, Moreno-Mondéjar et al. (2020) found that the diversity of eco-innovations was positively associated with firms’ sales growth.

Additionally, cooperation with universities has a significant and positive direct effect to the development of environmental innovations (coefficient 0.244). The greater the importance the firm places on cooperation with this particular agent, the wider the range of eco-innovations the firm develops. Therefore, our second hypothesis is supported, and we can conclude that value cocreation with universities leads to the development of eco-innovations. This finding is consistent with the argument that university-firm interaction, as well as knowledge collaboration with other non-business agents, increases firm’s capacity for environmental innovation (Dangelico et al., 2013; Jones and Zubielqui, 2017).

Our third hypothesis is not supported, because no significant relationship is found between cooperation with universities and operational flexibility. We expected that through cooperation with universities firms would acquire knowledge to make them able of making rapid changes in product design to a wide range of products and to meet an increasing variety of customer expectations. However, universities and research institutions do not seem to be a clear knowledge source for this particular capability.

Contrary to our expectations, we found no direct significant effect of cooperation with universities and performance. Hence, we cannot corroborate our fourth hypothesis. Similarly, no significant relationship was found of operational flexibility on eco-innovation. Then our fifth hypothesis is not supported.

On the contrary, operational flexibility positive and significantly affects performance (coefficient 0.204). Hence, our sixth hypothesis is corroborated, and we can state that the greater the firm’s capacity to make rapid changes in product design, rapidly adjust its production capacity, and offer a wide range of personalized products, the greater its performance, as measured in terms of increase in sales and benefits and/or cost reductions. This is particularly important in the food and beverage industry, because it allows firms to rapidly adjust to changes in consumer demands (Avermaete et al., 2004).

Operational flexibility reflects the firm’s capacity to face and respond to market dynamism and refers to having “built-in procedures which permit a high degree of variation in sequencing, scheduling, etc.” (Carlsson, 1989, p. 186). Hence, this capability would help firms to reduce environmental uncertainty (Alpkan et al., 2007). Operational flexibility allows firms to have the necessary flexibility to change production volumes and diversify product features to meet customer demand, thus enhancing performance (Zhang et al., 2014).

In regard to our control variable, we found a positive and significant effect (coefficient 0.167) on performance. Our findings are similar to those of previous literature (Navaretti et al., 2014; Jové-Llopis and Segarra-Blasco, 2018; Moreno-Mondéjar et al., 2020) that highlight the importance of company size on the association between eco-innovation and firm performance. Size is associated with firms’ resources and capabilities that enable them to develop the necessary knowledge base to promote eco-innovations (Segarra-Oña et al., 2013). The connection of company size with profitability is mainly based on the existence of economies of scale and/or market power (Fernández et al., 2019).

To summarize, firm performance is explained by cooperation with universities, eco-innovation, operational flexibility, and firm size. Although the explanatory power is not very high, we can partially explain firm performance based on these four variables (*R* ^2^ = 14.2%). In addition, this eco-innovative behavior significantly depends on firms’ cooperation with universities and operational flexibility (*R* ^2^ = 7.4%). Table 2 also shows the decomposition of the parameters of the model. It shows the total effect of the pathways between the variables as well as its direct, total indirect, and partial indirect effect. Particularly, it shows that, although there is no direct and significant effect of cooperation with universities on performance, there is a significant indirect effect (coefficient 0.064). Hence, the total effect is positive and significant (coefficient 0.140). The effect of cooperation with universities on performances is mainly mediated by eco-innovation.

Additionally, and as we have already mentioned, a firm’s performance is also influenced by its operational flexibility (coefficient 0.205, significant at 95%) and by its size (coefficient 0.177, significant at 95%).

## Conclusion

In this paper, we examine how cooperating with universities and research institutions may foster the development of value-added environmental innovations that improve firms’ performance. In doing so, we develop a model of the relationships between cooperation with universities, operational flexibility, eco-innovation, and firm performance. We test the model, using SEM, on a sample of 250 firms operating in the Spanish food and beverage industry.

Our findings show that firms that value their cooperation with universities develop a wider variety of environmental innovations and, through this, increase their performance. Eco-innovations are usually developed with different objectives, such as production efficiency or meeting environmental standards, in mind. This multi-purpose nature of goals may require knowledge from different sources. Cooperating with universities and research institutions helps firms to gather this knowledge. Previous literature argues that eco-innovative activities necessitate more external knowledge sources than other innovations (Horbach et al., 2013). Additionally, in regard to the effect on performance, our results are consistent with Cainelli et al. (2011) who found complementarity between eco-product and eco-process innovations. Similarly, Cheng et al. (2014) found that the best performers implemented more than one type of green practice.

These results have several implications for practitioners and policy makers. The former should be aware of the complementarities between different types of eco-innovative activities and how they may increase firm sales and reduce costs. Also, the wider the variety of environmental activities developed by the firm, the greater the benefits. Additionally, universities and research institutions are ideal partners for gathering the necessary external knowledge to develop this particular type of innovation. Administrators and policy makers should note that policies that foster cooperation with universities and research institutions will be more effective in achieving the goal of reducing corporate environmental harms.

Finally, several limitations of this paper should be acknowledged and taken into account when generalizing the results. The main limitation arises from the fact that our sample is country-specific and limited to a single industry. Future research should apply similar models to other industries and geographical contexts for comparison and/or generalization of the findings.

## Data Availability Statement

The datasets presented in this article are not readily available because Funding has been used for the use of the data. Requests to access the datasets should be directed to juanjaime.arroyave@alu.uclm.es.

## Author Contributions

All authors listed have made a substantial, direct and intellectual contribution to the work, and approved it for publication.

### Conflict of Interest

The authors declare that the research was conducted in the absence of any commercial or financial relationships that could be construed as a potential conflict of interest.
